# RNAseq Reveals Complex Response of *Campylobacter jejuni* to Ovine Bile and *In vivo* Gallbladder Environment

**DOI:** 10.3389/fmicb.2017.00940

**Published:** 2017-05-29

**Authors:** Amanda J. Kreuder, Jennifer A. Schleining, Michael Yaeger, Qijing Zhang, Paul J. Plummer

**Affiliations:** ^1^Department of Veterinary Diagnostic and Production Animal Medicine, College of Veterinary Medicine, Iowa State UniversityAmes, IA, United States; ^2^Department of Veterinary Microbiology and Preventative Medicine, College of Veterinary Medicine, Iowa State UniversityAmes, IA, United States; ^3^Department of Veterinary Pathology, College of Veterinary Medicine, Iowa State UniversityAmes, IA, United States

**Keywords:** *Campylobacter*, RNAseq, transcriptome, *in vivo* model, gallbladder, bile

## Abstract

Colonization of the gallbladder by enteric pathogens such as *Salmonella typhi, Listeria monocytogenes*, and *Campylobacter jejuni* is thought to play a key role in transmission and persistence of these important zoonotic agents; however, little is known about the molecular mechanisms that allow for bacterial survival within this harsh environment. Recently, a highly virulent *C. jejuni* sheep abortion (SA) clone represented by the clinical isolate IA3902 has emerged as the dominant cause for sheep abortion in the United States. Previous studies have indicated that the *C. jejuni* clone SA can frequently be isolated from the gallbladders of otherwise healthy sheep, suggesting that the gallbladder may serve as an important reservoir for infection. To begin to understand the molecular mechanisms associated with survival in the host gallbladder, *C. jejuni* IA3902 was exposed for up to 24 h to both the natural ovine host *in vivo* gallbladder environment, as well as ovine bile *in vitro*. Following exposure, total RNA was isolated from the bile and high throughput deep sequencing of strand specific rRNA-depleted total RNA was used to characterize the transcriptome of IA3902 under these conditions. Our results demonstrated for the first time the complete transcriptome of *C. jejuni* IA3902 during exposure to an important host environment, the sheep gallbladder. Exposure to the host environment as compared to *in vitro* bile alone provided a more robust picture of the complexity of gene regulation required for survival in the host gallbladder. A subset of genes including a large number of protein coding genes as well as seven previously identified non-coding RNAs were confirmed to be differentially expressed within our data, suggesting that they may play a key role in adaptation upon exposure to these conditions. This research provides valuable insights into the molecular mechanisms that may be utilized by *C. jejuni* IA3902 to colonize and survive within the inhospitable gallbladder environment.

## Introduction

*Campylobacter jejuni* is currently the leading cause of ovine campylobacteriosis in the United States (Wu et al., 2013), recently surpassing *C. fetus* subsp. *fetus* as the primary causative agent of bacterial abortion in sheep (Kirkbride, 1993; Delong et al., 1996). This change has been driven by the rapid emergence of a highly virulent sheep abortion (SA) clone that harbors chromosomally encoded tetracycline resistance and a unique porA gene that has been demonstrated to be essential in causing the abortion phenotype (Sahin et al., 2008; Wu et al., 2016). Outbreaks of zoonotic transmission to humans related to raw milk consumption have been reported (Sahin et al., 2012), highlighting the need for greater understanding of the mechanisms used by this highly virulent strain of *C. jejuni* to both cause disease and persist in animal hosts.

Chronic colonization and subsequent shedding of organisms into the environment is thought to play a key role in maintenance of *C. jejuni* in the sheep population. Abattoir studies of sheep and other ruminants have shown that the gallbladder is frequently positive for *C. jejuni* even in the absence of clinical disease (Ertas et al., 2003; Acik and Cetinkaya, 2006; Sahin et al., 2012). In order to decrease colonization and chronic shedding with *C. jejuni* in animal reservoirs, there is a critical need to understand the molecular mechanisms employed by this organism to survive exposure to bile and establish colonization of the gallbladder mucosa.

Although multiple *in vitro* studies have shown expression of key virulence factors in the presence of bile salts (Gaynor et al., 2001; Lin et al., 2003, 2005b; Raphael et al., 2005; Fox et al., 2007; Malik-Kale et al., 2008; Dzieciol et al., 2011), the concentrations of bile salts assessed in these studies were equivalent to physiologically relevant intestinal concentrations and were not representative of the much higher concentrations representative of intraluminal gallbladder conditions. Little is known about how *Campylobacter* adapts to the harsh environment of the gallbladder; however, the ability to survive in bile is likely critical to their survival and colonization of the rest of the gastrointestinal tract (Gunn, 2000). In addition to a basic lack of studies replicating gallbladder bile exposure *in vitro*, the use of *in vitro* studies alone does not fully capture the intricacies of the *in vivo* gallbladder environment, nor the ongoing interaction between host and bacteria that is likely to be encountered under conditions of natural infection.

Only three studies to date have been published assessing the *in vivo* transcriptome of *C. jejuni* under exposure to any host environment. Two of the three used microarray technology to assess transcriptional changes, and both determined that there are marked differences in gene expression profiles between *in vivo* and *in vitro* samples (Stintzi et al., 2005; Woodall et al., 2005). While microarray studies have been very useful in beginning to understand gene expression and regulation, they are limited in that they can only identify changes in known genes. The third *in vivo Campylobacter* transcriptome study published to date utilized the emerging technology of high throughput RNA sequencing (RNAseq) to assess the *in vivo* transcriptome of *C. jejuni* during colonization of the chick intestinal tract. Using this technology, the authors were able to demonstrate differential expression of both protein coding genes as well as identify numerous putative regulatory RNAs that were previously unknown (Taveirne et al., 2013). The rapid advancement of high throughput deep sequencing technologies along with the ability to assess the entire transcriptome without prior knowledge of genome structure has allowed RNAseq to quickly become the new method of choice for studying global gene expression (Croucher and Thomson, 2010; van Vliet, 2010; van Opijnen and Camilli, 2013). The power of global transcriptome studies utilizing the RNAseq approach to rapidly increase the knowledge base related to a particular area of interest is immense, and also is currently the method of choice for identification of the novel class of gene expression regulators, small non-coding RNAs (ncRNA, sRNA) (Sharma and Vogel, 2009). Using RNAseq technology, a large number of previously unknown non-coding RNAs have already recently been identified in other strains of *C. jejuni* (Chaudhuri et al., 2011; Butcher and Stintzi, 2013; Dugar et al., 2013; Porcelli et al., 2013; Taveirne et al., 2013); thus far the small RNA repertoire of sheep abortion clone IA3902 remains uncharacterized.

The overall goal of this study was to utilize RNA sequencing technology to study the transcriptome of *C. jejuni* IA3902 following exposure to both the *in vivo* gallbladder of a natural host species (sheep), as well as ovine bile *in vitro*. We reasoned that assessing exposure to both bile *in vitro* and the sheep gallbladder *in vivo* would enable the most complete assessment of the complex gene expression and regulatory networks necessary for survival within the host gallbladder environment provided to date. Specifically, we hypothesized that by utilizing the *in vivo* host environment we would be able to identify an increased number of candidate genes required for survival in the gallbladder environment when compared to utilization of an *in vitro* model of bile alone. In addition, we hypothesized that the newly identified class of regulators, non-coding RNAs, could be identified utilizing this same approach and would be observed to display changes in expression under the conditions studied.

By utilizing strand-specific total RNA sequencing on the Illumina HiSeq platform, we were able to identify 434 protein coding genes that were upregulated and 102 downregulated in the *in vivo* host environment following 24 h of exposure. In addition, 89 known and putative non-coding RNA genes were observed to be upregulated with 12 downregulated under the same conditions. The number of genes identified in the *in vivo* host environment was demonstrated to be almost twice the number identified at the same time points *in vitro*. This research provides for the first time valuable insights into gene expression patterns and potential regulatory mechanisms that may be employed by the highly virulent *C. jejuni* IA3902 to colonize and survive within the inhospitable gallbladder environment where it potentially serves as a chronic nidus of infection for spread of disease between animals and humans.

## Materials and methods

### Bacterial strains and culture conditions

A clinical isolate of the *C. jejuni* SA (sheep abortion) clone, IA3902, was used for the entirety of this study. This isolate was obtained from a sheep abortion outbreak in Iowa in 2006 (Sahin et al., 2008) and clonal isolates of this strain have been identified from within the gallbladder of sheep in abattoir studies (Sahin et al., 2012). *C. jejuni* IA3902 was routinely grown in Mueller-Hinton (MH) broth or agar plates (Becton-Dickinson, Franklin Lakes, NJ) at 42°C under microaerophilic conditions with the use of compressed gas (55% O_2_, 10% CO_2_, 85% N_2_).

For preparation of the IA3902 inoculums, cultures were grown on MH plates overnight for 16 h until lawn growth was achieved. Plates were then washed with 1 mL of MH broth and the wash fluid was collected into sterile 50 mL conical vials. The volume contained in each vial was then standardized and 500 μl of the collected culture was removed and processed immediately for RNA protection as described below. An additional 100 μl was also removed for standard plate counts to determine actual inoculum in CFU/mL. The remaining inoculum was then centrifuged at 3,000 × g for 5 min to pellet the cells and the inoculum was resuspended in a minimal amount of MH broth for inoculation. The prepared inoculums were then placed under microaerophilic conditions and used within 3 h of preparation.

All samples of bile to be inoculated with *C. jejuni* IA3902 (either *in vivo* or *in vitro*) were processed identically to determine if culturable bacteria were present prior to inoculation. For the *in vivo* study, 1 mL of bile was removed from the gallbladder of all animals prior to inoculation of *C. jejuni* IA3902 using a sterile 3 mL syringe and 20 gauge 1″ needle; this sample was stored at 4°C for less than 2 h until processed. For the bile to be used for *in vitro* studies, the entire volume of collected bile was collected into 50 mL conical vials and stored at 4°C for less than 2 h until processed. All samples were screened for bacterial growth at 37°C on blood agar (TSA with 5% sheep blood; Remel, Lenexa, KS) under aerobic and anaerobic conditions (GasPak EZ Anaerobe Pouch System; Becton-Dickinson), as well microaerophilic growth on MH plates at 42°C. Of the *in vivo* inoculated animals, one animal did not have any appreciable bile in its gallbladder at the time of inoculation, therefore fecal culture on MH plates supplemented with Preston *Campylobacter* selective supplement (Oxoid, Hampshire, United Kingdom) and *Campylobacter* growth supplement (Oxoid, Hampshire, United Kingdom) according to the manufacturer's recommendations for isolation of *Campylobacter* from fecal sources was used instead to screen for intestinal carriage of *C. jejuni* as a proxy for gallbladder carriage.

Of the 7 animals that had bile that could be harvested from the gallbladder pre-inoculation, only 1 animal displayed any growth under the conditions studied; the remaining cultures were free of any bacterial colonization as detectable by these methods. The single animal that exhibited bacterial growth displayed a pure growth of colonies on MH agar at 42°C microaerophilic that was confirmed to be *C. jejuni* utilizing a MALDI-TOF mass spectrometry biotyper (Bruker Daltonics, Billerica, MA) for identification. Screening of the feces from the single animal that did not have bile for collection pre-inoculation did not reveal the presence of *C. jejuni*.

Following inoculation and incubation of both *in vivo* and *in vitro* samples, 100 μL from each bile sample as well as 100 μl of mucosal scraping was set aside and used to determine the viable CFU/mL following exposure via serial dilution onto MH plates and incubation at 42°C microaerophilic using the drop-plate method as previously described (Chen et al., 2003).

### *In vivo* exposure of *C. jejuni* IA3902 to the sheep gallbladder environment

All animal experiments were approved by the Iowa State University Institutional Animal Care and Use Committee (IACUC) prior to initiation and followed all appropriate animal care guidelines. Preliminary experiments utilizing one or two mixed breed female sheep obtained from local farms were performed to determine the best method to inoculate the gallbladder of sheep with *C. jejuni* and subsequently harvest enough viable bacteria for RNA isolation. The various methods studied included transcutaneous ultrasound guided inoculation, inoculation via laparoscopy, and full laparotomy with direct visualization of the gallbladder for inoculation. Of the options attempted, full laparotomy with and without placement of a stainless steel medium-large Hemoclip® designed for vessels up to 10 mm (Weck, Research Triangle Park, NC) over the common bile duct were the only options to be successfully performed in a single animal each. Rapid excretion of bile into the duodenum from the gallbladder was noted in the animal without Hemoclip® placement which precluded collection of adequate total RNA at the end of study, therefore, all future inoculations were performed via full laparotomy with placement of a Hemoclip® over the common bile duct.

For the primary study, eight adult female mixed breed sheep were obtained from two local farms with no known history of *C. jejuni* related abortions. The sheep were randomly divided into two groups, either 2 or 24 h incubation, via a random number generator (http://www.random.org).

The animals were fasted for 12 h prior to anesthetic induction. A jugular catheter was placed and patency maintained for the remainder of the study using heparinized saline flushes every 8 h. General anesthesia was obtained using an intravenous triple drip solution consisting of 500 mL guaifenesin 5% + 500 mg ketamine + 50 mg xylazine. Anesthesia was induced in 5 to 10 min by the rapid administration of 0.5 to 2 mL/kg of this solution and maintained at a rate of 2 mL/kg/h until the end of the procedure. Once fully anesthetized, the animals were placed in left lateral recumbency and the right paracostal region was clipped and aseptically prepared for surgery.

Entry into the abdomen was made via a right paracostal approach to allow for best visualization of and access to the gallbladder. Following visualization of the gallbladder, the common bile duct was located and a Hemoclip® was placed to prevent outflow of bile from the gallbladder following inoculation. Using a sterile 3 mL syringe and 20 gauge 1″ needle, 1 mL of bile was removed from the gallbladder of all animals prior to inoculation of *C. jejuni* IA3902. Following removal of the pre-inoculation bile sample, 1.5 mL of MH broth containing approximately 10^11^ CFU of *C. jejuni* IA3902 inoculum was then injected into the lumen of the gallbladder using a separate 3 mL syringe and 20 gauge 1″ needle. The body wall incision was closed and the animals recovered uneventfully from surgery. Food and water were provided following recovery from anesthesia and animals were monitored for signs of pain or septicemia following the procedure.

At either 2 or 24 h post-inoculation as previously determined via random assignment, the sheep were humanely euthanized via intravenous injection of 1 mL/10 lb body weight pentobarbital (Fatal Plus®; Vortech, Dearborn, MI). Immediately following euthanasia, a clean incision was made into the ventral midline of the abdomen to expose the liver and gallbladder. Using a 16 gauge 1″ sterile needle and a 60 mL syringe, the entire amount of bile retained in the gallbladder was removed via gentle aspiration. The collected bile was immediately processed for RNA protection and isolation as described below and 100 μL was used for a serial dilution in MH broth to determine viable counts of *C. jejuni* (CFU/mL) following exposure to bile.

### *In vitro* bile inoculation and incubation

To compare the *in vivo* gallbladder environment to bile-only exposure *in vitro*, fresh bile was collected at necropsy from an additional group of eight sheep obtained from one of the same farms as above that were being used for an unrelated study. Again using a 16 gauge 1″ sterile needle and a 60 mL syringe, the entire amount of bile retained in the gallbladder was removed via gentle aspiration at the time of necropsy. Following collection, the bile was cultured as described above to determine if it was free of culturable bacteria. While awaiting culture results, the bile was stored at 4°C in sterile 50 mL conical tubes. Following confirmation of culture negative status, the entire collected amount of bile from four of the animals confirmed to be culture-negative (ranging in volume from 14 to 33 mL) was pre-warmed to ovine body temperature (39.5°C) in an incubator for 20 min and then inoculated with 10^11^ *C. jejuni* IA3902 suspended in MH broth prepared as described above for the *in vitro* study. Following inoculation, the bile was then incubated under microaerophilic conditions at 39.5°C in a static incubator to best replicate *in vivo* host conditions. At 2 h, the bile was mixed via gentle shaking and half of the total amount was removed for RNA protection and isolation as described below. The remaining bile was then incubated until 24 h at which time it was also processed for RNA protection and isolation in an identical manner.

### RNA extraction and DNase treatment

The bacterial inoculum samples that were set aside during preparation were processed immediately for RNA protection to maintain integrity of the RNA transcripts present. To minimize the number of replicates necessary for sequencing yet maintain a representation of all of the inoculums used, the inoculums for both the *in vivo* and *in vitro* experiments were pooled in sets of two for processing (total of four sets of samples from the *in vivo* and two sets of samples from the *in vitro*). As all of the inoculums were grown and prepared in an otherwise identical fashion, these six RNA samples were considered to represent six biological replicates of RNA expression by *C. jejuni* immediately prior to exposure to the tested environments. The inoculum samples were centrifuged at 8,000 × g for 2 min immediately following collection to rapidly pellet the cells while minimizing the time elapsed between collection and introduction of an RNA protection solution. Following pelleting of the cells, the supernatant was decanted and 1 mL QIAzol Lysis Reagent (QIAGEN, Germantown, MD) was added to the cultures to quench further RNA production and protect the RNA present from degradation. To resuspend the pellet, the mixture was pipetted up and down and vortexed at high speed for 1 min. Following vortexing, the QIAzol-culture mixture was incubated at room temperature for 5 min. QIAzol-protected cultures were then stored at −80°C for up to 2 months prior to proceeding with total RNA isolation.

For the bile samples inoculated with *C. jejuni* IA3902, immediately following collection from either the *in vivo* gallbladder or from the samples incubated *in vitro*, the bile was transferred into 15 mL conical tubes (FisherScientific) with no more than 7 mL bile per tube. The tubes were then centrifuged at 8,000 × g for 2 min to rapidly pellet the cells while minimizing the time elapsed between collection and introduction of an RNA protection solution. The bile supernatant was then decanted and QIAzol Lysis Reagent to add to the tube for RNA protection. For concentrated bile samples (less than 10 mL total recovered bile), 1 mL of QIAzol Lysis reagent was added to the cell pellet per 1.5 mL of the starting bile amount. For dilute bile samples (greater than 10 mL total recovered bile), 1 mL QIAzol was added per 3 mL of the starting bile amount. To resuspend the pellet, the mixture was pipetted up and down and vortexed at high speed for 1 min. Following vortexing, the QIAzol-culture mixture was incubated at room temperature for 5 min. QIAzol-protected cultures were then stored at −80°C for up to 2 months prior to proceeding with total RNA isolation.

Total RNA isolation was performed using the miRNeasy Mini Kit (QIAGEN) according to the manufacturer's instructions to isolate total RNA >18 nt. One column was used per 1 mL of QIAzol used for RNA stabilization. On-column DNase treatment was performed using the RNase-free DNase set (QIAGEN). Further treatment of 10 μg of extracted RNA was performed using the TURBO DNA-*free* kit (Life Technologies, Carlsbad, CA) following RNA isolation to remove any residual DNA contamination. The total RNA was then purified using the RNeasy MinElute Cleanup kit (QIAGEN) with the following modifications as recommended by QIAGEN Technical Services to retain total RNA, including RNA < 200 nt in length: (1) no more than 50 μL of RNA sample was used to enter the RNeasy MinElute Cleanup protocol at a time; (2) to the RNA sample, 350 μL of Buffer RLT was added, followed by 600 μL of 100% ethanol. The RNA-RLT-ethanol mixture then proceeded with the standard bind/wash/elute steps of the protocol as provided by the manufacturer.

RNA concentration was measured using the NanoDrop ND-1000 spectrophotometer (ThermoScientific, Wilmington, DE) and Qubit RNA BR Assay (ThermoFisher Scientific, Waltham, MA) and RNA quality was measured using the Agilent 2100 Bioanalyzer RNA 6000 Nano kit (Agilent Technologies, Santa Clara, CA). Verification of complete removal of any contaminating DNA was performed via PCR amplification of a portion of the CjSA_1356 gene, which is part of the capsule locus and has previously been determined via comparative genomics to only be present in *C. jejuni* IA3902, using primers SA1356F, and SA1356R (Luo et al., 2012). A single 24 h *in vivo* sample failed to isolate any RNA following extraction and purification; therefore, it did not continue with the rest of the library preparation.

### RNAseq library preparation and sequencing

Analysis of the *in vivo* collected RNA samples via the Agilent Bioanalyzer suggested that some samples likely contained host (ovine) RNA along with bacterial RNA; therefore, an rRNA removal kit suited to removal of both eukaryotic and prokaryotic rRNA was chosen for preparation of the RNAseq library. A total of 2.5 μg of confirmed DNA-free total RNA was treated with the Ribo-Zero Magnetic Gold rRNA Removal Kit (Epidemiology) according to the manufacturer's instructions (Illumina). Following rRNA removal, the rRNA-depleted RNA was again purified using the RNeasy MinElute Cleanup kit using the same modifications as described above. Following clean-up, the RNA was eluted into 12 μL of sterile RNase-free water; quality, quantity, and rRNA removal efficiency was then analyzed via the Agilent 2100 Bioanalyzer RNA 6000 Pico kit (Agilent Technologies).

Library preparation for sequencing on the Illumina HiSeq platform was completed using the TruSeq stranded mRNA HT library preparation kit (Illumina) with some modifications. As this kit was designed for use with eukaryotic RNA with poly-A tails, the initial poly-A RNA purification step was omitted. To enter the protocol, 5 μL of the rRNA-depleted RNA totaling approximately 200 ng was added to 13 μL of the “Fragment, Prime, Finish” mix. The remainder of the library preparation was carried out according to the manufacturer's instructions and all 24 samples were barcoded using the high-throughput (HT) 96-well RNA Adapter Plate (RAP) as supplied by the manufacturer. Following enrichment of the cDNA fragments, the quality of the cDNA was validated using the Agilent 2100 Bioanalyzer DNA 1000 kit (Agilent Technologies) and quantity was determined via the Qubit dsDNA BR Assay (ThermoFisher Scientific). Following library validation, the indexed cDNA samples were submitted to the Iowa State University DNA Facility where they were normalized and pooled according to the manufacturer's instructions. The pooled library was then sequenced on an Illumina HiSeq 2500 machine in high-output single read mode with 100 cycles.

### Differential gene expression analysis of RNAseq data

To analyze the differences in gene expression between the plate grown inoculum, *in vivo* gallbladder, and *in vitro* bile exposed strains *C. jejuni* IA3902 at various time points, Rockhopper (http://cs.wellesley.edu/~btjaden/Rockhopper/), a freely available RNAseq analysis platform, was used as previously described using the standard settings of the program (McClure et al., 2013). Using this program, results of gene expression are normalized and reported by the program as reads per kilobase per million reads (RPKM), with the exception that instead of dividing by the total number of reads, Rockhopper divides by the upper quartile of gene expression.

Following computational analysis via Rockhopper, a change in gene expression was deemed significant when the *Q*-value (false discovery rate) was below 5% and a >1.5-fold change in expression levels was present. If in any condition being compared the expression level (RPKM) was “0,” it was changed to “1” to allow for statistical analysis to be performed. Any significant changes in 16S or 23S rRNA genes were ignored as these were determined to be due to differences in efficiency of rRNA removal by Ribo-Zero and not inherent differences between strains and conditions. Read count data was visually assessed using the Integrated Genome Viewer (IGV) (https://www.broadinstitute.org/igv/) (Robinson et al., 2011; Thorvaldsdottir et al., 2013). Differentially expressed genes were then assessed for function using the Clusters of Orthologous Groups (COG) (Galperin et al., 2015) as previously described in IA3902 (Wu et al., 2013) with statistical significance calculated using hypergeometric probability testing (https://www.geneprof.org/) (Halbritter et al., 2011). Venn diagrams depicting overlap of genes differentially regulated in multiple conditions were generated using the Venny website (http://bioinfogp.cnb.csic.es/tools/venny/index.html) (Oliveros, 2007-2015). Metabolic pathway analysis was performed using the Kegg Pathways when appropriate (http://www.genome.jp/kegg/pathway.html) (Kanehisa et al., 2015).

## Results

### Summary of strand-specific RNAseq results

**Figure 1:**
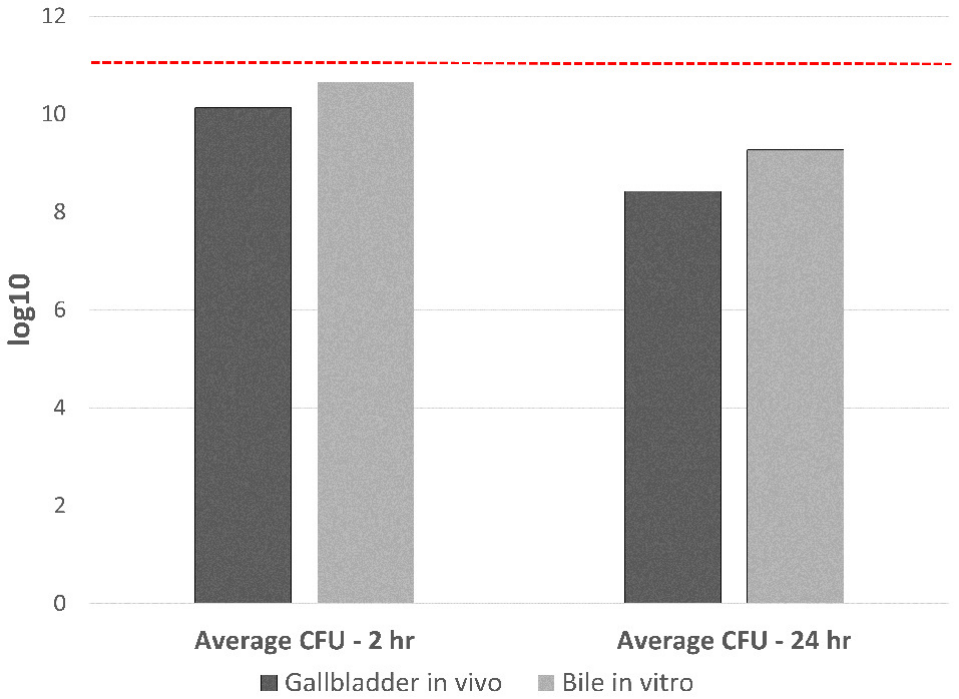
**Total CFU of ***C. jejuni*** IA3902 recovered following 2 and 24 h exposure to either the ***in vivo*** gallbladder environment or sheep bile only ***in vitro*****. Standard plate counts were used to determine the initial starting inoculum amount which averaged 10^11^ total CFU for all *in vivo* and *in vitro* studies (indicated in the figure as a dashed red line). Standard plates counts were again used to determine the CFU/mL of viable *C. jejuni* following both *in vivo* and *in vitro* incubation for 2 or 24 h; this was then mutiplied by the total volume of bile collected to determine the total CFU of bacteria recovered. An average was then taken from the four replicates for each condition at each time point to demonstrate amount of starting material for RNA extraction under the different conditions.

Figure 1 demonstrates the inoculated amount of *C. jejuni* IA3902 compared with the average amount of viable bacteria present following incubation of either 2 or 24 h in both ovine bile *in vitro* and ovine gallbladders *in vivo*. Despite reduction in total number of bacteria following incubation, total RNA of sufficient quality and quantity was able to be extracted from all but a single 24 h *in vivo* gallbladder sample. Overall, 21 barcoded libraries were sequenced in a single lane on the Illumina HiSeq 2500 yielding over 74 million reads, with close to 67 million high quality reads aligning to either the genome or pVir plasmid of *C. jejuni* IA3902 and averaging 3,176,824 reads per library (Supplementary Table 1). The majority of reads (average of 72% of total reads), mapped to protein coding genes of the chromosome, with an average of 20% of reads mapping to ribosomal RNA following rRNA depletion with Ribo-Zero (median of 15%). Only seven of the 21 libraries contained less than or equal to 10% ribosomal RNA reads, which would be consistent with the manufacturer's predicted rRNA removal efficiency. The majority of the libraries (14 of 21) exhibited less efficient rRNA removal (>10% rRNA reads) with one library completely failing to exhibit rRNA removal at all (93% of reads mapped to rRNA genes). An average of 1% of reads mapped to antisense regions of the annotated protein coding genome on both the chromosome and pVir plasmid. On the pVir plasmid, 91% of reads mapped to protein coding genes, while an average of 8% of reads were to unannotated regions.

### Differential gene expression analysis of RNAseq data

A summary of the differences in numbers of genes with increased and decreased expression identified via Rockhopper under either *in vivo* or *in vitro* bile exposure when compared to unexposed IA3902 is given in Table 1. Overall, the *in vivo* samples consistently identified a larger number of genes when compared to the same time point *in vitro*. In addition, a larger number of genes were identified at the 24-h time point when compared to 2 h both *in vivo* and *in vitro*. For the 24-h time point, 86 operons of the 363 predicted by Rockhopper to exist on both the IA3902 chromosome (350) and pVir plasmid (13) demonstrated at least 2 consecutive genes differentially upregulated, with 21 of those exhibiting changes in all of the genes predicted in the operon. Conversely, 21 of the 363 predicted operons (19 chromosomal, 3 pVir) were demonstrated to have at least 2 consecutive genes downregulated, with 9 of those exhibiting changes in all of the genes predicted in that operon. Supplementary Tables 2–5 list all of the genes that were determined to be differentially expressed when compared to the unexposed IA3902 inoculum for each of the *in vivo* and *in vitro* conditions and time points. Supplementary Table 2, which lists all of the genes differentially expressed at the 24-h *in vivo* timepoint also includes annotation to indicate which genes are part of predicted multi-gene operons.

**Table 1:** **Summary of the differences in numbers of genes with increased and decreased expression under either ***in vivo*** or ***in vitro*** bile exposure when compared to unexposed IA3902 at both 2 and 24 h**.

	**Condition**							
	***In vivo*** **gallbladder**				***In vitro*** **bile**			
	**2 h**		**24 h**		**2 h**		**24 h**	
	**Chr**	**pVir**	**Chr**	**pVir**	**Chr**	**pVir**	**Chr**	**pVir**
**Protein-coding genes**								
Decreased expression	105	7	96	6	10	4	54	7
Increased expression	283	10	420	14	102	7	248	11
**Non-coding RNA genes**								
Decreased expression	[15]	[1]	[12]	[0]	[7]	[0]	[16]	[1]
Increased expression	[62]	[1]	[87]	[2]	[44]	[1]	[53]	[1]

Chr, IA3902 chromosome;

pVir, pVir plasmid associated with IA3902;

[], *signifies that this is a putative list generated by Rockhopper of predicted non-coding RNA as well as known non-coding RNA genes*.

**Figure 2:**
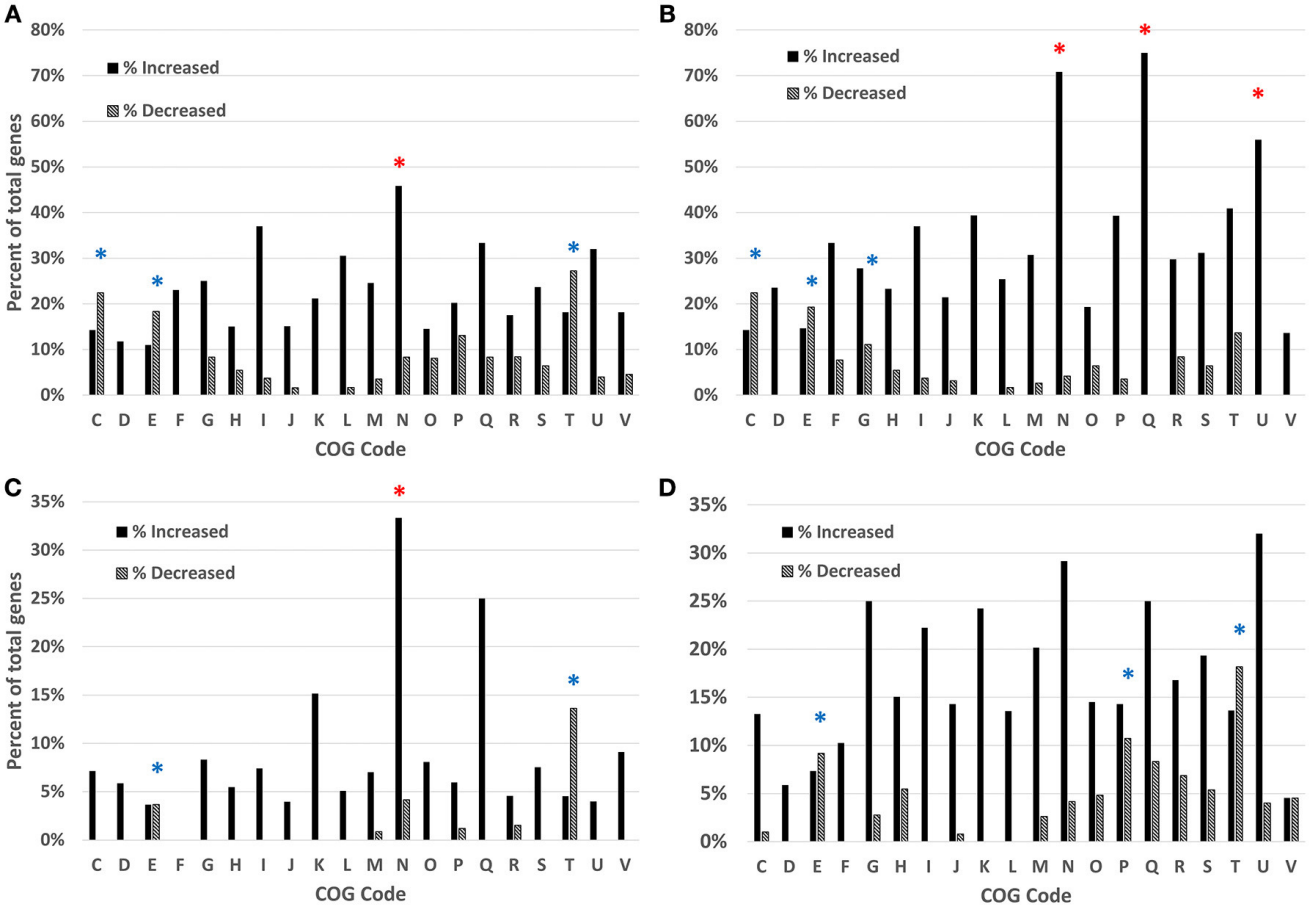
**Analysis of the differentially expressed genes based on COG function detected by RNAseq, gallbladder ***in vivo*** samples at 2 h (A)** and 24 h **(B)** and bile *in vitro* samples at 2 h **(C)** and 24 h **(D)**. Clusters of Orthologous Groups (COG) categories are indicated on the x-axis, with the percentage of genes enriched shown on the y-axis. Black bars indicate increased expression, patterned bars indicate decreased expression. Red asterisks (^*^) indicate statistically significant enrichment (*p* < *0.05)* for genes with increased expression; blue asterisks (^*^) indicate statistically significant enrichment (*p* < *0.05)* for genes with decreased expression. COG category codes: C, Energy production and conversion; D, Cell cycle control, mitosis and meiosis; E, Amino acid transport and metabolism; F, Nucleotide transport and metabolism; G, Carbohydrate transport and metabolism; H, Coenzyme transport and metabolism; I, Lipid transport and metabolism; J, Translation; K, Transcription; L, Replication, recombination and repair; M, Cell wall/membrane biogenesis; N, Cell motility; O, Posttranslational modification, protein turnover, chaperones; P, Inorganic ion transport and metabolism; Q, Secondary metabolites biosynthesis, transport and catabolism; R, General function prediction only; S, Function unknown; T, Signal transduction mechanisms; U, Intracellular trafficking and secretion; V, Defense mechanisms; W, Extracellular structures.

To estimate the functional categories of genes affected by each condition and time point, the COG function for each gene was mapped and the totals compiled for each condition and time point. These totals were then compared to the total number of possible genes within each category in *C. jejuni* IA3902 and the percentage of possible genes differentially expressed was then calculated for each category along with the hypergeometric probability to test for significance (Figure 2). For all conditions and time points studied, the “cell motility” category demonstrated either the highest or second highest percentage of total genes upregulated with statistically significant (*p* < 0.01) enrichment in three of the four conditions. “Secondary metabolites biosynthesis, transport and catabolism” and “intracellular trafficking and secretion” were also highly upregulated on a percentage basis with statistically significant enrichment (*p* < 0.02) for both in the *in vivo* 24 h environment. “Cell wall/membrane biogenesis” was consistently noted to be the top category represented in all conditions and time points based on total number of genes upregulated rather than percentage but did not reach statistical significance. In contrast, the categories observed to have the highest percentage of genes exhibiting decreased expression in all comparisons and time points were “amino acid transport and metabolism,” which exhibited statistically significant enrichment (*p* < 0.02) in all four conditions, as well as “signal transduction mechanisms” which showed statistically significant enrichment (*p* < 0.01) in three of the four conditions studied.

**Figure 3:**
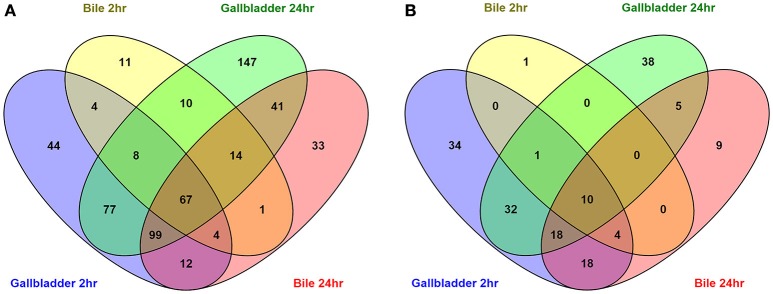
**Venn diagrams depicting the overlap of upregulated (A)** and downregulated **(B)** annotated genes in all conditions compared to the unexposed *IA3902* inoculum. Differential expression of all known annotated genes were compared to each other utilizing the Venny program.

To compare the upregulated annotated genes that were identified for all four conditions with the unexposed IA3902 inoculum, a Venn diagram was constructed to allow for visual comparisons (Figure 3A). A total of 67 known genes were found to be upregulated in all 4 conditions, suggesting that these genes are required for survival following exposure to bile regardless of *in vivo* or *in vitro* exposure (Table 2). Of particular interest within the observed genes upregulated under all conditions examined, two hypothetical proteins (CjSA_0040 and CjSA_0528) exhibited extreme upregulation when compared to plate grown control samples; this response was particularly robust in the host environment for both genes. CjSA_0040 is predicted to be a 107 amino acid protein that appears to be well conserved across the *Campylobacter* genus but is not found in other genera of bacteria. Assessment for conserved structural domains was performed using NCBI Protein BLAST and yielded no predictions of conserved structure for CjSA_0040. Rockhopper predicted that CjSA_0040 belongs to the same operon as *flgD* and *flgE*, therefore, it is reasonable to suggest that CjSA_0040 is also likely involved in motility. CjSA_0528 is predicted to be a 309 amino acid protein that also appears to be well conserved across the *Campylobacter* genus but again the sequence does not appear to be conserved in other genera of bacteria. Assessment for conserved structural protein domains was performed using NCBI Protein BLAST and in this case did yield a prediction of conserved structure within the outer membrane channel domain for CjSA_0528. Proteins within this family are considered to be part of the porin superfamily and may be related to gram negative porins or ligand gated channels (Marchler-Bauer et al., 2015). Investigation of other strains of *C. jejuni* revealed that CjSA_0528 is a homolog of the putative periplasmic protein Cj0561c in strain 11168. Expression of Cj0561c has previously been shown to be strongly repressed by CmeR and transcription of Cj0561c induced in the presence of bile salts (Guo et al., 2008).

**Table 2:** **List of annotated genes identified to be upregulated or downregulated under all four conditions when compared with the unexposed IA3902 inoculum**.

**Name**	**Product**	**Fold change**			
		**GB 2 h**	**GB 24 h**	**Bile 2 h**	**Bile 24 h**
**UPREGULATED GENES**					
CJSA_0035	MFS family drug resistance transporter	3.5	3.3	2.0	3.7
CJSA_0040	Hypothetical protein	21.4	52.2	10.9	15.1
CJSA_0041	Hypothetical protein	3.1	6.1	1.6	2.2
CJSA_0045	Putative iron-binding protein	2.8	5.3	3.8	4.2
CJSA_0049	Hypothetical protein	1.9	3.9	1.5	1.6
CJSA_0056	Hypothetical protein	3.0	10.6	1.6	3.2
*folk*	Hydroxymethyldihydropteridine pyrophosphokinase	2.7	3.3	2.9	3.1
*atpF'*	F0F1 ATP synthase subunit B	2.7	4.1	1.9	2.5
CJSA_0126	Hypothetical protein	2.5	4.4	2.3	2.9
CJSA_0166	Putative lipoprotein	4.8	12.4	2.2	3.5
CJSA_0305	Hypothetical protein	2.8	4.9	1.5	2.8
*cmeB*	RND efflux system, inner membrane transporter CmeB	3.7	1.7	1.7	1.9
*cmeA*	RND efflux system, membrane fusion protein CmeA	3.8	2.6	2.3	2.1
CJSA_0370	Hypothetical protein	3.2	6.6	2.6	5.0
*sdhA*	Succinate dehydrogenase, flavoprotein subunit	6.5	2.4	2.5	1.5
*sdhB*	Succinate dehydrogenase, iron-sulfur protein subunit	5.0	2.0	2.5	1.5
CJSA_0426	Hypothetical protein	3.5	10.4	1.8	3.8
CJSA_0528	Hypothetical protein	29.3	21.3	17.3	15.8
*Nth*	Endonuclease III	2.2	3.9	2.3	4.2
CJSA_0596	Prophage Lp2 protein 6	1.5	2.4	1.5	2.1
CJSA_0636	Hypothetical protein	2.2	2.8	1.5	2.3
*flgH*	Flagellar basal body L-ring protein	1.6	4.4	2.0	1.7
CJSA_0655	Hypothetical protein	2.4	4.9	1.6	3.9
CJSA_0716	Hypothetical protein	2.9	2.8	1.7	1.7
CJSA_0785	Hypothetical protein	2.8	9.7	2.3	6.2
CJSA_0818	Hypothetical protein	2.7	1.6	2.7	2.2
*dsbB*	Putative disulfide oxidoreductase	3.5	1.8	2.6	2.2
*flgL*	Flagellar hook-associated protein FlgL	2.2	5.3	1.9	1.9
CJSA_0852	Hypothetical protein	1.9	4.2	1.5	2.0
CJSA_0920	Hypothetical protein	2.0	3.7	2.3	1.5
*rpoD*	RNA polymerase sigma factor RpoD	1.7	2.9	1.7	1.9
*cmeE*	Membrane fusion component multidrug efflux system CmeDEF	1.9	2.0	1.5	1.5
CJSA_0977	Adenylosuccinate lyase	2.5	5.2	2.4	3.4
*Rho*	Transcription termination factor Rho	1.8	1.9	1.6	1.7
CJSA_1129	Putative PAS domain containing signal-transduction sensor	4.8	5.7	1.8	2.1
CJSA_1146	Putative 5-formyltetrahydrofolate cyclo-ligase family protein	1.6	4.1	1.6	2.3
CJSA_1180	Hypothetical protein	2.1	7.1	1.9	2.1
*pseC*	C4 aminotransferase specific for PseB product	2.0	3.1	1.7	1.5
CJSA_1233	Hypothetical protein	2.7	3.9	1.6	1.7
*maf1*	Motility accessory factor	2.3	3.3	2.4	2.2
*neuC2*	Putative UDP-N-acetylglucosamine 2-epimerase	4.4	6.0	1.8	2.5
CJSA_1266	Hypothetical protein	1.6	4.1	1.6	2.3
*maf4*	Motility accessory factor	2.5	3.5	2.3	2.3
*nrfA*	Putative periplasmic cytochrome C	3.4	3.8	3.2	3.3
*nrfH*	Putative periplasmic cytochrome C	6.1	11.1	7.8	6.3
CJSA_1304	Putative nucleotide phosphoribosyltransferase	1.8	1.5	1.5	2.1
CJSA_1387	Hypothetical protein	2.1	5.0	1.7	1.8
*infA*	Translation initiation factor IF-1	2.9	2.9	1.7	2.3
CJSA_1533	Hypothetical protein	1.8	2.2	1.9	2.2
CJSA_1544	Hypothetical protein	2.9	6.7	3.1	3.8
CJSA_1554	Hypothetical protein	1.8	2.3	1.5	2.2
CJSA_1562	Hypothetical protein	2.6	4.5	1.5	2.1
CJSA_1596	Putative MSF family efflux protein	3.9	4.0	3.1	4.0
CJSA_1624	GNAT family acetyltransferase	3.0	7.3	1.7	2.5
CJSA_pVir0024	Putative plasmid partioning ParA protein	1.7	4.1	4.2	4.5
CJSA_pVir0025	Hypothetical protein	4.6	5.4	4.2	5.3
*repE*	Putative replication protein RepE	3.4	10.9	4.3	6.5
CJSA_pVir0040	Hypothetical protein	3.5	8.1	2.5	3.9
CJSA_pVir0044	Hypothetical protein	5.4	22.4	6.0	13.8
CJSA_t0001	Ala tRNA	6.1	8.0	2.2	3.4
CJSA_t0002	Ile tRNA	8.1	9.4	1.9	3.2
CJSA_t0004	Ala tRNA	5.0	5.9	1.5	2.1
CJSA_t0014	Ala tRNA	4.6	6.3	2.0	2.5
CJSA_t0015	Ile tRNA	7.7	8.3	1.7	3.0
CJSA_t0023	Val tRNA	2.2	4.7	1.7	1.5
CJSA_t0043	Ala tRNA	1.9	3.8	1.6	1.9
CJSA_t0044	Val tRNA	2.3	4.9	1.8	2.0
**DOWNREGULATED GENES**					
CJSA_0025	Sodium/dicarboxylate symporter	−7.2	−3	−2.4	−7.6
*cjaA*	Putative amino-acid transporter periplasmic solute-binding	−5.3	−4.2	−3.5	−3.7
*metF*	5,10-methylenetetrahydrofolate reductase	−2.7	−2.2	−4.2	−6.3
*putP*	Putative sodium/proline permease	−4.7	−9	−2.0	−2.9
CJSA_1573	ABC transporter permease	−2.1	−1.9	−2.9	−7
CJSA_pVir0008	Hypothetical protein	−4.2	−11.7	−2.5	−2.7
CJSA_pVir0009	Hypothetical protein	−3.8	−10.6	−2.4	−2.7
CJSA_pVir0050	Hypothetical protein	−6.7	−7.5	−4.1	−5.3
CJSA_pVir0051	Hypothetical protein	−5	−5	−4.4	−5
CJSA_t0030	Ser tRNA	−7.4	−4.2	−3.6	−6.9

*A comparison of fold change under each condition is provided*.

Bile, Bile only in vitro condition;

*GB, Gallbladder in vivo condition*.

An additional 125 genes were identified that were upregulated in 3 of the 4 conditions; the likelihood that these genes are also important to the response to bile is high. Conversely, a total of 10 genes (Table 2) were observed to be downregulated in all 4 conditions when compared to unexposed IA3902 again utilizing a Venn diagram for visual comparison (Figure 3B), with an additional 23 genes observed to be downregulated in 3 of the 4 conditions.

**Figure 4:**
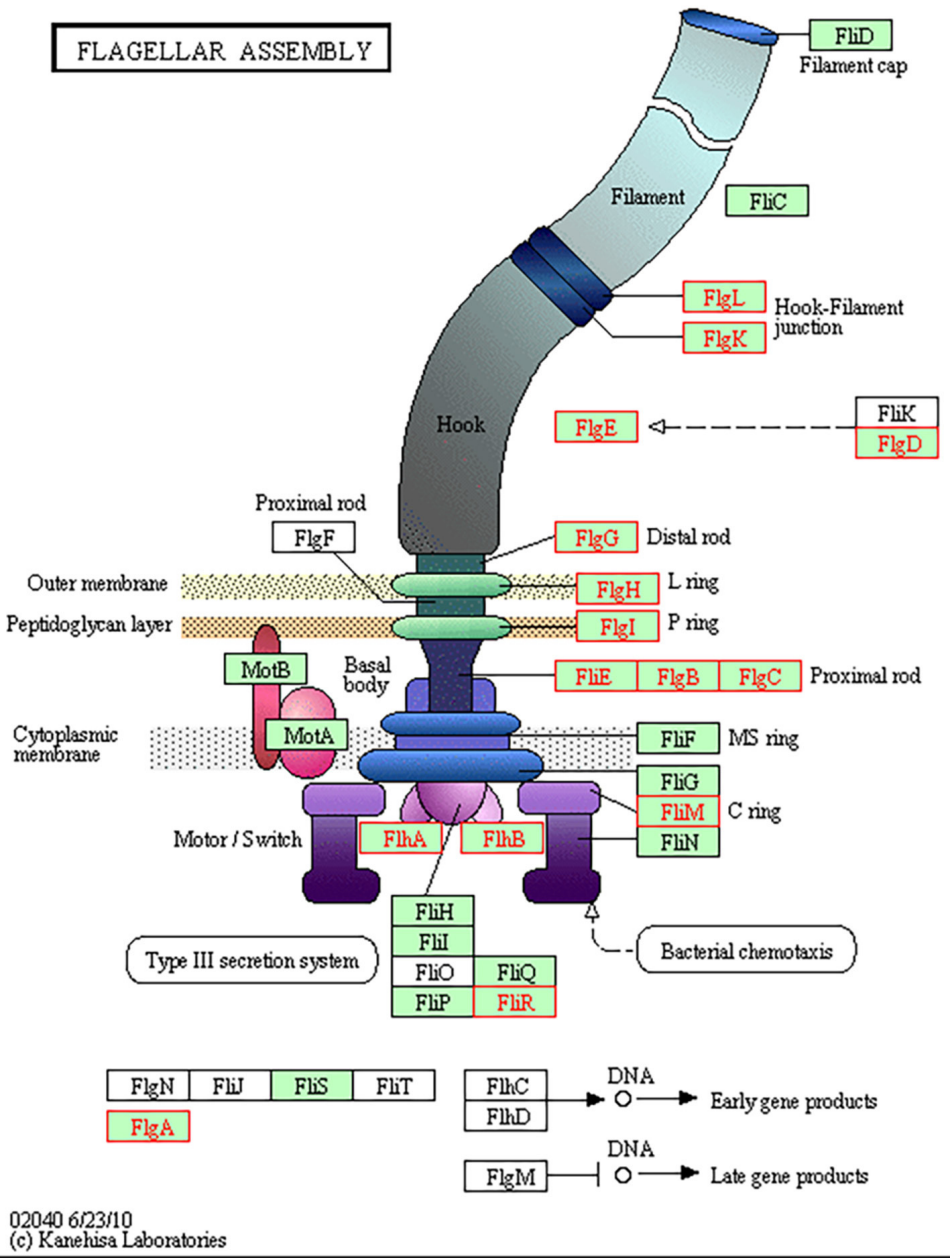
**KEGG Pathway for flagellar assembly in ***C. jejuni***.** Multiple genes responsible for flagellar assembly were observed to be upregulated in the gallbladder condition only; image depicts 24 h in the *in vivo* gallbladder environment (increased differential expression denoted by red highlight).

Table 3 demonstrates the 77 genes that were identified to be only upregulated in both *in vivo* conditions, suggestive of a role related to sensing of and interaction with the host environment unique from exposure to bile alone. Multiple genes responsible for chemotaxis were observed to be upregulated in both *in vivo* conditions only, including *cheY, cheR*, and a putative methyl-accepting chemotaxis protein (MCP) CjSA_0897. The CheA-CheY phosphor-relay pathway has previously been shown to act as the master switch to control taxis by altering the direction of flagellar rotation from a swimming phenotype (counter-clockwise rotation) to a tumbling phenotype (clockwise rotation) (Lertsethtakarn et al., 2011). The *cheY* gene has also been shown to be required for adhesion and invasion (Yao et al., 1997). CjSA_0897 is a putative methyl-accepting chemotaxis protein (MCP); MCPs have been shown to play an important role in sensing the environmental signals to activate the CheA-CheY system (Lacal et al., 2010). As there would be minimal signals present in the *in vitro* environment from the host to direct chemotaxis nor host cells to adhere to or invade, it seems reasonable that these important genes would only be upregulated in the host environment where seeking out other host locations would be advantageous. The ability to seek out new environments relies heavily on cell motility, which was demonstrated to be an overall area of increased gene expression in our data. Figure 4 demonstrates genes upregulated in the Kegg pathway for flagellar assembly at 24 h in the *in vivo* gallbladder environment. While *flaA* was not observed to be upregulated in our data, the increased expression of these additional flagella-associated genes suggests that increased flagellar assembly is occurring.

**Table 3:** **List of annotated genes identified to be upregulated only under the ***in vivo*** gallbladder 2 h and gallbladder 24 h conditions and not in bile alone at either timepoint when compared with the unexposed IA3902 inoculum; a comparison of fold change under each condition is provided**.

**Name**	**Product**	**Fold change**	
		**GB 2 h**	**GB 24 h**
*dsbI*	DsbB family disulfide bond formation protein	2.1	2.7
*Dba*	Disulfide bond formation protein	3.0	6.5
*fliM*	Flagellar motor switch protein FliM	1.9	2.6
*fliA*	Flagellar biosynthesis sigma factor	2.0	4.4
*cydA*	Cytochrome d ubiquinol oxidase, subunit I	2.4	3.1
CJSA_0080	Putative lipoprotein	1.8	2.3
*obgE*	GTPase ObgE	1.5	3.4
CJSA_0111	Putative recombination protein RecO	5.4	5.6
CJSA_0112	Putative metalloprotease	3.1	1.9
*thrB*	Homoserine kinase	2.2	2.0
CJSA_0149	Cytochrome c family protein	1.5	1.6
CJSA_0150	Putative 6-pyruvoyl tetrahydropterin synthase	1.8	1.9
CJSA_0228	Hypothetical protein	1.6	1.7
CJSA_0233	Sulfatase	2.9	2.5
*dgkA*	Diacylglycerol kinase	3.6	4.9
*pyrC*	Dihydroorotase	2.3	2.8
CJSA_0240	Zinc transporter ZupT	2.5	2.1
*mreC*	Rod shape-determining protein MreC	1.8	1.6
*lpxB*	Lipid-A-disaccharide synthase	1.6	1.7
*Ndk*	Nucleoside diphosphate kinase	1.8	1.9
*flhB*	Flagellar biosynthesis protein FlhB	1.9	2.4
CJSA_0327	Ppx/GppA family phosphatase	1.8	1.7
*fdxB*	Ferredoxin, 4Fe-4S	1.6	1.9
CJSA_0358	Integral membrane protein	1.5	1.6
CJSA_0384	GTP-binding protein	2.7	2.6
CJSA_0424	Hypothetical protein	3.1	2.7
CJSA_0425	Hypothetical protein	2.5	3.3
*rpmG*	50S ribosomal protein L33	1.5	2.3
CJSA_0467	Hypothetical protein	1.8	1.7
CJSA_0474	Gfo/Idh/MocA family oxidoreductase	2.2	2.3
CJSA_0517	Hypothetical protein	2.1	1.7
CJSA_0572	Putative polyphosphate kinase	1.7	1.8
CJSA_0575	ABC-type transmembrane transport protein	1.9	2.0
*recN*	DNA repair protein RecN	1.5	1.7
CJSA_0699	Hypothetical protein	3.1	4.9
CJSA_0717	Hypothetical protein	2.6	2.9
*purU*	Formyltetrahydrofolate deformylase	1.5	2.2
*ftsK*	Putative cell division protein	2.1	2.8
CJSA_0894	Peptidyl-arginine deiminase family protein	2.1	1.7
CJSA_0897	Putative HAMP containing membrane protein	2.0	2.0
CJSA_0910	Putative acyl-CoA thioester hydrolase	2.2	2.7
*flgR*	Sigma-54 associated transcriptional activator	1.7	5.2
CJSA_0968	Hypothetical protein	2.1	5.3
*Ssb*	Single-stranded DNA-binding protein	1.6	1.8
*mfd*	Transcription-repair coupling factor	1.9	1.9
CJSA_1086	Hypothetical protein	3.1	5.3
CJSA_1118	ABC transporter ATP-binding protein	2.2	3.9
CJSA_1153	Peptidase, M23/M37 family	1.8	1.5
*kefB*	Putative glutathione-regulated potassium-efflux system	1.5	2.5
*uvrC*	Excinuclease ABC subunit C	3.3	4.4
CJSA_1190	Hypothetical protein	1.6	2.1
*racS*	Two-component sensor (histidine kinase)	1.5	2.0
*ktrA*	Putative K+ uptake protein	1.9	1.7
*hisF*	Imidazole glycerol phosphate synthase subunit HisF	2.3	1.5
*maf3*	Motility accessory factor	2.1	1.7
CJSA_1305	Putative periplasmic protein (VacJ-like protein)	1.6	1.5
CJSA_1416	Hypothetical protein	1.9	2.4
*selD*	Putative selenide, water dikinase	1.7	1.9
CJSA_1448	Hypothetical protein	1.8	2.6
*rpsM*	30S ribosomal protein S13	1.7	1.7
*rnhA*	Ribonuclease H	1.7	1.7
*murL*	Glutamate racemase	2.5	2.5
*nlpC*	Putative lipoprotein nlpC	1.7	1.8
*nhaA2*	Na+/H+ antiporter	1.7	3.1
CJSA_1631	Putative GTP cyclohydrolase I	2.6	13.9
CJSA_pVir0016	Hypothetical protein	2.9	2.6
CJSA_pVir0020	Hypothetical protein	2.0	1.8
CJSA_t0009	Thr tRNA	1.7	2.9
CJSA_t0011	Arg tRNA	2.3	2.7
CJSA_t0012	Met tRNA	1.6	4.4
CJSA_t0020	Val tRNA	1.8	3.6
CJSA_t0033	Leu tRNA	1.8	1.8
CJSA_t0034	Cys tRNA	1.7	3.4
CJSA_t0035	Ser tRNA	2.3	4.2
CJSA_t0037	Arg tRNA	1.9	1.9
CJSA_t0038	Arg tRNA	2.1	1.9
CJSA_t0040	Pro tRNA	1.5	1.7

*GB, Gallbladder in vivo condition*.

Table 4 demonstrates a summary of the number of genes found to be differentially expressed when the 2 and 24 h time points for each condition were compared. Again, a greater number of genes were observed to be increased at the 24 h time point as opposed to the 2 h time point *in vivo*, suggesting continued evolution of the response to changes in the host environment over time. In contrast, very few genes were observed to be substantially different between the 2 and 24 h time points *in vitro*. As the *in vitro* environment was static between the time points, it appears reasonable to suggest that little further adaptation was necessary between 2 and 24 h for survival within bile alone.

**Table 4:** **Summary of the number of genes found to be differentially expressed when the 2 and 24 h time points for each condition were compared to each other**.

	**Condition**	
	**2 vs. 24 h**	
	**GB**	**Bile**
**Protein-coding genes**		
Increased at 2 vs. 24 h	38	16
Increased at 24 vs. 2 h	136	9
**Non-coding RNA genes**		
Increased at 2 vs. 24 h	[4]	[8]
Increased at 24 vs. 2 h	[57]	[3]

*GB, Gallbladder in vivo condition*.

*Bile, Bile only in vitro condition*.

### Identification of differentially expressed putative non-coding RNAs

**Figure 5:**
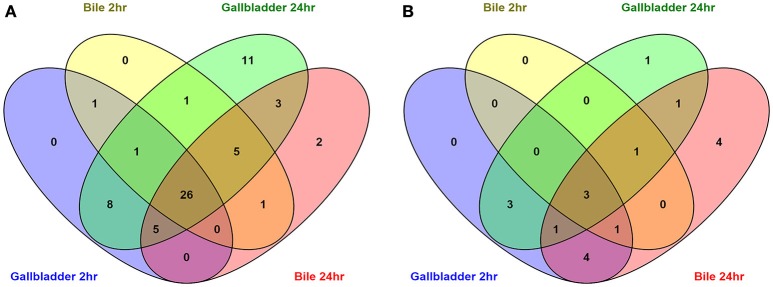
**Venn diagrams depicting the overlap of upregulated (A)** and downregulated **(B)** predicted non-coding RNA unannotated genes in all conditions compared to the unexposed *IA3902* inoculum. All putative ncRNA predicted by Rockhopper as differentially expressed under each condition were compared to each other utilizing the Venny program.

A total of 91 potential non-coding RNA were predicted by the Rockhopper program, with 27 of the predictions indicating an antisense RNA, and the other 64 predictions being small RNAs primarily located within intergenic regions. Differential expression of these predicted non-coding RNAs was performed identically to the known annotated genes by the Rockhopper program and the complete results are included in the previously described Supplementary Tables 2–5. Figure 5 demonstrates a Venn diagram which allows visual comparison of all of the putative non-coding RNAs that were identified for all four conditions when compared to unexposed IA3902. A total of 26 predicted non-coding RNAs were found to be upregulated in all 4 conditions, as well as 3 noted to be downregulated in all conditions (Supplemental Table 6). Manual examination of the entire list of non-coding RNAs predicted by Rockhopper did identify several previously validated non-coding RNAs (Dugar et al., 2013) as present and differentially regulated in our dataset; the genomic location of these ncRNAs is listed in Table 5 along with their differential expression between conditions in Table 6. Manual evaluation of the transcriptomic data in IGV also identified transcripts present in the locations of the following previously identified ncRNAs predicted to exist in IA3902 by Dugar et al. (2013): CjNC10, CjNC110, CjNC170, CjNC190, CjNC200, and tracrRNA, as well as reads antisense to CjSA_0158 (CJas_0168c), CjSA_0336 (CJas_0363c), CjSA_0668 (CJas_0704). Rockhopper failed to identify these transcripts as putative ncRNA candidates, however, likely due to lower levels of expression under the conditions studied.

**Table 5:** **List of previously validated ***C. jejuni*** non-coding RNAs (Dugar ***et al***., 2013) expressed and differentially regulated in our dataset with location, length, and orientation**.

**Transcription start-stop**	**Length**	**Strand**	**Product**	**LFG**	**RFG**	**Orientation**
**INCREASED EXPRESSION**						
250059-249960	99	−	CjNC20	CjSA_0232	CjSA_0233	<<<
675392-675240	152	−	CjNC60	CjSA_0681	*dnaE*	><>
1155464-1155580	116	+	CjNC120	*groEL*	*dccS*	>><
1193304-1193433	129	+	CjNC140	CjSA_1197	*porA*	>>>
1572367-1572461	94	+	CjNC180	CjSA_1562	*Map*	>><
25244-25412	168	+	CJpv2	CjSA_pVir0032	CjSA_pVir0032	>><
**DECREASED EXPRESSION**						
1183928-1183941	13	+	CjNC130/6S	CjSA_1188	CjSA_1188	>>>

*All ncRNAs identified were located within the intergenic regions of the left flanking (LFG) and right flanking (RFG) genes*.

LFG, left flanking gene;

RFG, right flanking gene;

<<<or >>>, flanking genes and ncRNA expressed in the same direction;

*><> or >><, flanking genes and ncRNA expressed in different directions*.

**Table 6:** **Differential expression observed under each condition of previously validated non-coding RNAs (Dugar et al., 2013)**.

**Product**	**Fold change**			
	**GB 2 hr**	**GB 24 hr**	**Bile 2 hr**	**Bile 24 hr**
**INCREASED EXPRESSION**				
CjNC20	1.1	**1.5**	**2.8**	**1.7**
CjNC60	0.8	1	1.3	**1.7**
CjNC120	1.3^a^	**3.8**	1.1^a^	1.2^a^
CjNC140	**3.1**	**9.9**	1.4^a^	**4.6**
CjNC180	**6**	**19.8**	**1.8**	**4.8**
CJpv2	−1.4	**2.6**	−1.2	−1.1
**DECREASED EXPRESSION**				
CjNC130/6S	−**5.7**	−**3.5**	−1.8^b^	−1.8^b^

a *Q value >0.05 but fold change < 1.5*.

b *fold change >1.5 but Q value < 0.05*.

Bold indicates significant differential expression

GB, Gallbladder in vivo condition;

*Bile, Bile only in vitro condition*.

## Discussion

The rapid advancement of high throughput deep sequencing technologies along with the ability to assess the entire transcriptome without prior knowledge of genome structure have allowed RNAseq to become the new method of choice for studying gene expression (Croucher and Thomson, 2010; van Vliet, 2010; van Opijnen and Camilli, 2013). With the rapid increase in the quality of RNASeq data over the past several years and the use of technical and biologic replicates, this next-generation sequencing approach will likely soon be thought of to have the same reliability as RT-PCR experiments, the current gold standard for gene expression evaluation (de Sa et al., 2015). While RT-PCR can only be utilized to assess gene expression one gene at a time, RNAseq allows for global transcriptome analysis. Thus, the power of RNAseq to rapidly increase the knowledge base related to a particular area of interest is immense. RNAseq is also currently the method of choice for identification of the novel class of gene expression regulators, small non-coding RNAs (ncRNA, sRNA) (Sharma and Vogel, 2009). Using RNAseq technology, a large number of previously unknown non-coding RNAs have already recently been identified in other strains of *C. jejuni* (Chaudhuri et al., 2011; Butcher and Stintzi, 2013; Dugar et al., 2013; Porcelli et al., 2013; Taveirne et al., 2013). The data generated in our study represents the first report of the small RNA repertoire of the emergent and highly virulent *C. jejuni* sheep abortion clone IA3902.

Overall, the dataset that was generated from this study provides a very robust assessment of the global transcriptome of *C. jejuni* within an important and relevant natural host environment. The method of RNA isolation used was able to maintain high quality total RNA despite the challenges associated with RNA extraction from bile. The use of strand-specific RNA sequencing on the Illumina HiSeq platform following rRNA depletion using Ribo-Zero yielded adequate numbers of high quality reads that successfully aligned to the genome of IA3902 albeit with a higher than anticipated number of reads mapping to the rRNA genes. The rRNA depletion kit utilized, Ribo-Zero Magnetic Gold rRNA Removal Kit (Epidemiology), is specifically designed for use with eukaryotic (human/mouse/rat) and prokaryotic (gram positive and negative bacteria) mixed samples as would be encountered during *in vivo* experiments. This kit was chosen due to visual evidence of potential eukaryotic RNA presence observed in the RNA samples post-isolation, however, the drawback to utilizing a mixed population epidemiology kit is that a decreased number of probes were likely present to target gram negative rRNA. As the majority of the rRNA still likely originated from gram negative bacteria in this case, it is likely that the binding capacity of those probes was exceeded and thus increased the amount of bacterial rRNA that remained in the sample despite the visual appearance of removal of all 16S and 23S rRNA via the Agilent Bioanalyzer. The overall percentage of reads that mapped to the *C. jejuni* genome was quite high, indicating that very little eukaryotic or other types of prokaryotic RNA were present in the samples. Only one sample failed to rRNA deplete, while an additional sample was observed to have a lower percentage of reads mapping to *C. jejuni* IA3902. Analysis of differential gene expression via Rockhopper with and without the inclusion of these samples yielded minimal alterations in results; therefore, it was elected to maintain the samples within the dataset. Despite the minor difficulties related to less than ideal rRNA depletion, the samples averaged over 3 million high quality mapped reads per sample. Previous studies undertaken to assess the necessary amount of reads per prokaryotic sample to generate statistically significant data indicate that when data from well-correlated biological replicates are included, 2–3 million reads per sample enables a significant number of genes differentially expressed to be identified with high statistical significance (Haas et al., 2012). By utilizing the Ribo-Zero depletion, a much higher percentage of reads were able to be mapped to genes other than those rRNA-associated, making the overall use of this technique advantageous for this study.

One of the primary goals in generating this data was to identify expression of small non-coding RNAs which may play a role in the ability of *C. jejuni* to adapt and survive within bile and the gallbladder environment. Non-coding RNAs can be rapidly produced as they do not require translation to be active, and once produced in the cell they can rapidly be recycled if necessary (Papenfort and Vogel, 2010). Non-coding RNAs can also regulate multiple different targets within a cell in a variety of ways to coordinate rapid responses to changing environments (Waters and Storz, 2009). Based on the demonstrated ability of small RNAs to rapidly respond to changing environments and thus rapidly mediate altered translation of genes, it seemed reasonable that small RNAs should play a key role in bacterial adaptation to bile exposure and the *in vivo* gallbladder. Analysis of expression data by Rockhopper and prediction of non-coding RNAs demonstrated expression of a number of previously identified non-coding RNAs (7 identified by Rockhopper, with 9 additionally identified by manual curation) that were validated to exist in the closely related *C. jejuni* 11168 and predicted to exist in IA3902 based on sequence homology (Dugar et al., 2013). In contrast, of the predicted small RNAs to have >80% nucleotide identity to regions within the IA3902 genome in Dugar et al. (2013), 9 were not observed to have any transcription in the region of homology. This is consistent with observations in both *Campylobacter* as well as other species of bacteria such as *Listeria* where expression of conserved ncRNAs has been shown to be very divergent even among closely related strains (Wurtzel et al., 2012; Dugar et al., 2013). Differences in growth temperatures, library construction protocols, and prediction algorithms have also been shown to play a role in the ability to detect non-coding RNAs between separate experiments even within the same strain (Taveirne et al., 2013), thus these nuances also likely played a role in this case.

Of the seven ncRNAs confirmed to be observed in IA3902 by Rockhopper in our data, all exhibited differential gene expression, with six of the seven demonstrating increased expression in at least one of the conditions studied. CjNC140 and CjNC180 demonstrated differential expression in the majority of conditions and time points studied (three of four, and all four, respectively) and were observed to be consistently increased to a greater degree in the *in vivo* rather than *in vitro* conditions. This suggests that these non-coding RNAs may play a key role in the ability of *C. jejuni* to sense the changing host environment and respond quickly to those changes. The exact mechanism by which these small RNAs exert their regulatory control cannot be determined at this time as it is quite possible that each could, for example, serve to both stabilize some mRNA transcripts for increased protein expression while at the same time targeting other mRNA transcripts for degradation and decreased protein expression. Interestingly, expression in the region of CjNC190, a small RNA reported antisense to CjNC180 was also observed but again, not at high enough levels to be identified by the Rockhopper program. No reads were observed in the region of CjNC190 in the plate grown samples, with increased expression subjectively visible in all bile exposed conditions and time points, suggesting that both CjNC180 and CjNC190 may play a role either together or separately in adaptation to the bile environment. No additional publications describing the function of any of these small RNAs in *Campylobacter* have been published at this time; therefore, future work to elucidate the targets and mechanisms of action of these potent regulators is warranted.

Of the identified non-coding RNAs, the only one observed to be downregulated, particularly within the *in vivo* host conditions, was CjNC130, which has been demonstrated to be a 6S RNA homolog. The 6S RNA has been shown in other model organisms to play an important role in regulating transcription on a global scale by competing with DNA promoters for binding to RNA polymerase (Wassarman and Storz, 2000). The coding sequence of 6S is not conserved across bacterial genera, however, computational searches based on secondary structure have allowed for its identification across much of the prokaryotic kingdom (Wehner et al., 2014). The formation of a secondary structure consisting of a large double stranded hairpin with a central bulge is essential as it resembles an open promoter complex that allows for binding to RNA polymerase (Barrick et al., 2005). The exact function of CjNC130 in Campylobacter remains unknown at this time. Further work to understand the role of this newly identified non-coding RNA in regulation of global gene expression appears to be warranted.

The presence of antisense transcripts within datasets such as this has garnered great debate and discussion over the past few years (Sharma et al., 2010; Dugar et al., 2013; Conway et al., 2014). Preliminary data associated with this project but not included in this paper yielded 4% antisense reads, while the overall average of the data presented here demonstrates less than 1% of the total reads being antisense to known ORFs. RNA protection, extraction, and purification were identical between projects, however, newer rRNA depletion and library preparation methods were used in the study presented here. This suggests that some of the observed antisense reads may be a spurious artifact of RNA library preparation and that the strand specificity of RNA library preparation technology may have improved over the 3 year span between the preparation of the separate libraries. Further work to continue to improve the detection of true vs. spurious antisense transcripts within bacterial transcriptomes is warranted.

The dataset generated from this study also provides a very robust assessment of the global transcriptome of *C. jejuni*, allowing for identification of genes associated with survival in both bile and within the *in vivo* gallbladder of the natural ovine host. As the amount of bacteria inoculated into each condition was already higher than the concentration typically observed during *in vitro* growth under laboratory conditions in standard media, our differential expression dataset cannot be used to identify genes associated with growth of *C. jejuni* IA3902 in bile or in the gallbladder. A requirement of a false discovery rate (*Q* value) of less than 0.05 along with a fold change of greater than 1.5 was utilized to narrow the list of proposed differentially regulated genes to a robust, yet hopefully biologically relevant list. The fact that a large number of complete operons were observed to be differentially regulated in our data provides additional confidence that the results obtained are likely to be statistically sound and biologically relevant. In addition, analysis of the COG function of the differentially expressed genes was utilized to assess for enrichment of certain functional categories within the dataset. For all conditions and time points studied, the “cell motility” category demonstrated either the highest or second highest percentage of total genes upregulated and exhibited statistically significant enrichment in three of the four conditions. As motility has previously been demonstrated to be a requirement for *in vivo* colonization and virulence of *C. jejuni* (Guerry, 2007), it seems reasonable that an increased expression of genes associated with motility would be an important part of the response to the bile and gallbladder environments. In addition, it is highly likely that *C. jejuni* would seek out a more hospitable location within the gallbladder, such as the mucous layer and mucosal lining, for chronic colonization. This behavior has already been described for *C. jejuni* in the small intestine (McSweegan and Walker, 1986; Shigematsu et al., 1998), and to be able to achieve this requires effective motility.

“Secondary metabolites biosynthesis, transport and catabolism” and “intracellular trafficking and secretion” were also consistently upregulated under all conditions and demonstrated statistically significant enrichment under the *in vivo* 24 h condition, indicating that these functional categories may be important for survival particularly in the host. While not noted to be within the top categories on a percentage basis, “cell wall/membrane biogenesis” was consistently noted to be the top category represented in all conditions and time points based on total number of genes upregulated rather than percentage. Based on the strong detergent properties of bile salts that have been shown to be highly antibacterial as well as able to induce cellular lysis (Coleman et al., 1979; Begley et al., 2005), rapid repair and turnover of cell wall and membrane components is likely to be a key part of survival by *C. jejuni* when exposed to high concentrations of bile salts in the bile or in the gallbladder. In contrast, the categories observed to have the highest percentage of genes exhibiting decreased expression in all comparisons and time points were “amino acid transport and metabolism,” which exhibited statistically significant enrichment in all four conditions, as well as “signal transduction mechanisms” which showed statistically significant enrichment in three of the four conditions studied. On the surface, it appears counterintuitive that expression for these categories of genes be down regulated as it would be expected that there would be increased need for transmission of extracellular signals into the cell and increased amino acid turnover to provide for increased protein production. When assessed more closely, in all cases there were a similar number of genes also upregulated within the same categories, which suggests a shift in the specific pathways utilized for these cellular processes rather than an overall decrease in these important cellular processes.

Despite the importance of adaptation to bile exposure for all bacteria surviving within the gastrointestinal tract, very little published work has focused on the molecular mechanisms by which intestinal pathogens survive exposure to bile. The studies that have been previously performed in *C. jejuni* have focused on the concentrations of bile salts typically found in the intestinal tract (i.e., −1% w/v), not within the gallbladder itself (i.e., −10% w/v). As presented in Table 7, only 14 genes have been specifically reported to be involved in the bile tolerance response in *Campylobacter*, 2 of which are not present in strain IA3902. The efflux pumps *cmeABC* and *cmeDEF* are arguably the most important genes to have previously been shown to play an important role in resistance of *Campylobacter* to bile salts *in vitro* (Lin et al., 2002; Akiba et al., 2006). The observation of universally increased expression of *cmeAB* and *cmeE* in all conditions exposed to bile in our study when compared to plate growth confirms that these genes, while not expressed at high levels, are likely critical to survival when exposed to bile. As *cmeC* and *cmeF* are the last genes transcribed in each of the operons, it is reasonable to suggest that some decrease in the amount of full length transcript produced may occur as transcription moves across the operon, thus the reason that the fold change for those genes was always observed to be less than the corresponding gene at the start of the operon and significance was not reached in all conditions. Bile salts (cholate and taurocholate) have previously been show to induce expression of *cmeABC in vitro* in a time and dose dependent manner ranging from 6- to 16-fold increases in expression (Lin et al., 2005b). A much lower magnitude of increase was observed when exposed to pure bile, both *in vivo* and *in vitro*, in our study. This suggests that the complexities of complete bile may blunt the response observed when only certain components such as bile salts are assessed under controlled settings. The expression of *cmeDEF* has previously been noted to be intrinsically lower than *cmeABC*, and inactivation of *cmeF* has been demonstrated to increase expression levels of *cmeABC* (Akiba et al., 2006). While overall expression levels for both operons were similar in our study, this suggests that the efflux pumps encoded by *cmeABC* and *cmeDEF* may work together to ensure the viability of *Campylobacter* under conditions of exposure to toxic substances such as bile.

**Table 7:** **Fold change expression data for the 14 genes previously reported to be involved in the bile tolerance response in ***Campylobacter***, 2 of which are not present in strain IA3902**.

**Name**	**Present in IA3902**	**Fold Change**				**Expected change**	**References**
		**GB 2 h**	**GB 24 h**	**Bile 2 h**	**Bile 24 h**		
*cmeA*	Yes	**3.8**	**2.6**	**2.3**	**2.1**	Increase	Lin et al., 2002, 2005b
*cmeB*	Yes	**3.7**	**1.7**	**1.7**	**1.9**	Increase	Lin et al., 2002, 2005b
*cmeC*	Yes	**2.6**	**1.5**	1.2	**1.8**	Increase	Lin et al., 2002, 2005b
*cmeD*	Yes	**1.9**	**3.8**	1.4^a^	**2.6**	Increase	Akiba et al., 2006
*cmeE*	Yes	**1.9**	**2**	**1.5**	**1.5**	Increase	Akiba et al., 2006
*cmeF*	Yes	**1.9**	1.5	1.1	1.3	Increase	Akiba et al., 2006
*cmeR*	Yes	1.3^a^	1.1	1.2^a^	**1.7**	None	Lin et al., 2005b
*cbrR*	Yes	1.1	**1.9**	1.0	1.4^a^	None	Raphael et al., 2005
*ciaB*	Yes	0.8	1	0.9	1.4^a^	Increase	Rivera-Amill et al., 2001
*flaA*	Yes	0.9	1	1.3	0.7	Increase	Allen and Griffiths, 2001
*tlyA*	Yes	NE	NE	NE	NE	Increase	Malik-Kale et al., 2008
*dccR*	Yes	1.2	1.4^a^	1.0	1.4^a^	Increase	Malik-Kale et al., 2008
*hcp1*	No	−	−	−	−	−	Lertpiriyapong et al., 2012
*icmF1*	No	−	−	−	−	−	Lertpiriyapong et al., 2012

NE, not expressed under any conditions studied (<5 RMPK);

a *Q value > 0.05 but fold change < 1.5*.

*Bold indicates significant differential expression*.

GB, Gallbladder in vivo condition;

*Bile, Bile only in vitro condition*.

Expression of *cmeABC* has also been previously shown to be under the control of the transcription repressor CmeR. Exposure to bile salts *in vitro* has been shown to inhibit binding of CmeR to the promoter of *cmeABC* and allow for increased transcription of the *cmeABC* operon (Lin et al., 2005a). Exposure to cholate *in vitro* in the same study did not demonstrate an increased in expression of the *cmeR* gene. In our study, mild increases in *cmeR* expression were observed that were only found to be statistically significant *in vitro* at 24 h. Based on the previously described interaction of CmeR with bile salts, it is likely that these mild increases in expression have minimal biological effect on *cmeABC* expression as CmeR-mediated repression is likely to be inhibited under these conditions. The *cmeDEF* operon has been shown to be unaffected by CmeR repression (Akiba et al., 2006).

The response regulator CbrR (C*ampylobacter* bile response regulator) has also been shown to be required for resistance to the effects of bile salts, as mutants lacking it are unable to grow under sub-inhibitory concentrations of sodium deoxycholate (Raphael et al., 2005). It is believed that CbrR is a response regulator that is part of a two-component regulatory system which typically also includes a sensor kinase. Minimal changes in expression of this gene were noted in our data, with only the *in vivo* 24-h condition found to have a statistically significant increase in expression. Because of the proposed role as a response regulator, it seems reasonable that while its presence is necessary for survival when exposed to bile, its expression level may not need to change for its function to be fulfilled. The signal mediated by this system is likely to lead to downstream changes in the expression of multiple genes, however, that may affect the ability of *Campylobacter* to respond to exposure to bile.

The secretory protein CiaB (C*ampylobacter* invasion antigen B) has also been suggested to play a role in bile tolerance and has been demonstrated to be secreted upon co-cultivation of *C. jejuni* with intestinal cells and play a role in the ability of *C. jejuni* to invade host cells (Konkel et al., 1999). As synthesis and secretion of the CiaB protein have been demonstrated to be independent events, it has been proposed that *C. jejuni* normally begins to synthesize the Cia proteins upon passage into the small intestine, accumulates them within the cell, and then secretes them upon contact with the host cells lining the gastrointestinal tract as a concentrated release may be necessary to evoke an effect on the host cells (Rivera-Amill et al., 2001). Increased expression when exposed to the bile salt sodium deoxycholate *in vitro* was demonstrated via RT-PCR, which suggests that exposure to bile salts in the intestinal tract might normally be the trigger for increased expression (Rivera-Amill et al., 2001). Interestingly, expression of *ciaB* was not demonstrated to be significantly altered in any of the conditions in our study. A tendency toward decreased expression was noted at 2 h both *in vivo* and *in vitro*, with levels above non-exposed controls slightly increased at 24 h in both conditions. There are several possibilities to explain these findings. The response to the bile environment may have been very rapid and thus not present by the time samples were taken at 2 h, or it may have occurred during the time between the 2 and 24 h samples. Additional studies have shown that *ciaB* expression when exposed to deoxycholate was maximal at 12 h and began to decline again by 15 h (Malik-Kale et al., 2008). It is also possible that the higher levels of bile salts encountered in the gallbladder do not have the same effect as low concentrations such as what would be found in the intestinal tract. Finally, as these RNA samples were taken from bacteria free within the lumen of the gallbladder, and not intimately in contact with host cells, it is conceivable that direct contact with host cells may play a role in expression *in vivo*.

The last gene of interest to be previously described as important in the response of *Campylobacter* to exposure to bile is *flaA* (Alm et al., 1993), which is responsible for production of the FlaA protein, one of two protein subunits that form the flagellar filament. It has been previously demonstrated through the use of reporter fusions that the σ ^28^ promoter of *flaA* is upregulated when exposed to bovine bile, bile salts (deoxycholate), and L-fucose (Allen and Griffiths, 2001). In our study, expression of *flaA* was not shown to be statistically different under any of the conditions studied. This was an unexpected finding given the previous work done *in vitro*; however, as the *in vitro* work looked at very specific conditions and did not actually measure gene transcripts, only promoter activity, it is possible that exposure to a complex host environment renders a different response, or again, that the increased expression response was missed in the time points studied.

A few additional works have attempted to assess the response of *Campylobacter* to bile on a more global scale. Microarray analysis of RNA extracted from *C. jejuni* strain F3011 cultured with 0.1% deoxycholate for 12 h resulted in a total of 156 upregulated and 46 downregualted genes under these conditions (Malik-Kale et al., 2008). In addition to increased expression of the known bile-associated virulence genes *ciaB* and *cmeABC*, they also identified increased expression of two additional virulence factors: *dccR*, which has been shown to be part of a two-component system regulatory system that may play a role in the *in vivo* colonization ability of *C. jejuni* (MacKichan et al., 2004), and *tlyA*, a hemolysin that has been shown to be important for *Helicobacter* sp. *in vivo* colonization ability (Martino et al., 2001). Expression levels of *tlyA* were minimal to non-existent in all conditions examined in our work; this may be related to differences between strains of *C. jejuni*. Expression of *dccR* was observed to be increased 1.4-fold at 24 h under both *in vivo* and *in vitro* conditions; therefore, it is possible that during the time period between 2 and 24 h significantly increased expression may have occurred. In a separate study, Fox et al., 2007 used protein expression following 18 h of exposure to 2.5-5% oxbile added to rich media to identify 14 proteins with increased expression. Comparison of the proteins found to be increased to our work demonstrated no correlation with increased expression of the mRNA transcripts of those same exact proteins; however, some of the basic categories of upregulated genes were the same. While this previous work represents important information regarding exposure to differing levels of bile *in vitro*, it is possible that differences between the simplified *in vitro* environment and the complex *in vivo* environment presented in our study allowed for differing results. In addition, altered translation efficiency in the absence of increased presence of the mRNAs of the respective proteins may also play a role and lead to difficulty in comparing protein expression to transcriptomic data.

One of the biggest advantages, but also disadvantages, of generating RNAseq transcriptomic data under multiple *in vivo* and *in vitro* conditions as we have demonstrated here is the sheer amount of data that is generated. For the work presented here we have limited our analysis of the data to answering the original hypotheses of the study, however, many other comparisons and conclusions can likely be drawn from this data and will be the focus of future work.

## Conclusions

In summary, this is the first report of the complete transcriptome of *C. jejuni* IA3902 during exposure to an important and relevant natural host environment, the sheep gallbladder. We have demonstrated that the transcriptional “landscape” during direct interaction within the host, as displayed by utilizing *in vivo* inoculation of and RNA recovery from the sheep gallbladder environment, provides a more robust picture of the complexity of gene regulation required for survival when compared to *in vitro* exposure to ovine bile alone. A subset of genes were identified that are believed to play an important role in survival within bile, as well as survival in the host environment, including two highly expressed hypothetical proteins that warrant further study. In addition to the identification of important protein coding genes that are differentially expressed, seven previously identified non-coding RNAs were also confirmed to be differentially expressed within our data, suggesting that they may also play a key role in rapid regulation of gene expression upon exposure to bile and the host environment.

## Author contributions

AK and PP were responsible for the design, execution and data analysis of all material presented in this article. JS was responsible for performing the surgical inoculation of the sheep gallbladders. MY was responsible for necropsy and sample collection from the sheep gallbladder. QZ assisted with design and data interpretation.

### Conflict of interest statement

The authors declare that the research was conducted in the absence of any commercial or financial relationships that could be construed as a potential conflict of interest.

## Supplementary material

### Nucleotide sequence accession number

The RNA-seq sequencing data generated in this publication have been deposited in the NCBI Database under BioProject ID number PRJNA383351 (http://www.ncbi.nlm.nih.gov/bioproject/383351).
